# Characterizing circulating nucleosomes in the plasma of dogs with lymphoma

**DOI:** 10.1186/s12917-021-02991-x

**Published:** 2021-08-16

**Authors:** Christopher Dolan, Tasha Miller, Jarvis Jill, Jason Terrell, Theresa Kathleen Kelly, Thomas Bygott, Heather Wilson-Robles

**Affiliations:** 1 Small Animal Clinical Sciences Department, Texas A&M University, College of Veterinary Medicine, College Station, TX 77843 USA; 2 Volition America LLC, 13215 Bee Cave Parkway, Galleria Oaks B, Suite 125, Austin, TX 78738 USA; 3 Volition Diagnostics UK Ltd, 93-95 Gloucester Place, London, W1U 6JQ UK

**Keywords:** Canine, Lymphoma, Circulating nucleosomes, Cell free DNA, Circulating DNA, Histone 3.1

## Abstract

**Background:**

Nucleosomes consist of DNA wrapped around a histone octamer core like beads on a string so that DNA can be condensed as chromatin into chromosomes. Diseases such as cancer or inflammation lead to cell death where chromatin is fragmentated and released as mononucleosomes into the blood. The Nu.Q™ H3.1 assay measures total nucleosome concentration in plasma of humans and has been used to detect and identify cancer even at early stages. The objectives of this study were to determine if nucleosome levels could be used to distinguish between healthy dogs and dogs with various stages of lymphoma (LSA) using the Nu.Q™ H3.1 assay.

A total of 126 dogs diagnosed with LSA and 134 healthy controls were recruited for this study. Plasma was collected from each dog and stored in K2-EDTA tubes. The LSA patient samples were recruited from TAMU or purchased from various biobanks. All control cases were recruited from TAMU.

**Results:**

Dogs with LSA had an approximately 7-fold increase in their plasma nucleosome concentrations compared to controls (AUC 87.8%). Nucleosome concentrations increased with cancer stage and dogs with B cell lymphomas had significantly higher nucleosome concentrations than dogs with T cell lymphomas.

**Conclusions:**

The Nu.Q™ H3.1 assay was able to reliably detect elevated nucleosome concentrations in the plasma of dogs with LSA. Furthermore, it appears that nucleosomes are useful for differentiating cancer from healthy individuals in canines.

## Background

Liquid biopsy is a growing field in human medicine and has significant potential in veterinary medicine as it enables the use of minimally invasive techniques and analysis of tumor-derived material including circulating tumor cells, extracellular vesicles, and cell free DNA, among others. Information provided through these tools in cancer patients can provide early detection of neoplastic disease, provide prognostic information, monitor response to treatment, and help identify druggable targets [1, 2]. Furthermore, liquid biopsy assays are much more amenable to serial testing when compared to traditional tissue biopsies or expensive imaging tests.

Nucleosomes are the basic repeating subunit of chromatin consisting of DNA wrapped around a histone core [3]. Nucleosomes regulate several important functions within the cell in part due to a complex network of modifications and regulatory enzymes that control their positioning and stability. Due to the variety and flexibility of modifications, nucleosomes provide the framework for chromatin assembly, epigenetic regulatory mechanisms, while also protecting DNA from damaging agents [4].

Cell free DNA is released into the bloodstream, as nucleosomes, from a variety of cell types that are undergoing apoptosis or necrosis, but are most commonly released from hematopoietic cells as part of normal cellular turnover [5–7]. Low levels of cfDNA have been identified in healthy individuals and increased concentrations are present during various disease processes [8]. Nucleosomes have been shown to have different immunostimulatory potential as compared to circulating free histones and cfDNA [9]. Therefore, while these circulating components are related and share similar origins, they should be considered distinct entities with potentially different functions. Nucleosomes are elevated in humans and dogs with significant inflammation and increased nucleosome concentrations have been shown to be prognostic for survival in dogs experiencing trauma [10–12]. In neoplastic disease processes, nucleosomes are elevated in human patients with colorectal cancer and could help with early detection of this disease [13]. Nucleosomes have also been able to predict response to therapy in patients with advanced non-small-cell lung cancer [14]. Similarly, cfDNA levels are elevated in dogs with various tumor types and cfDNA levels correlated well with clinical stage [15, 16]. While nucleosomes themselves have not been extensively evaluated in canine neoplastic disease, a recent study did show significantly elevated nucleosome concentrations in a small cohort of dogs diagnosed with lymphoma (LSA) [17].

Lymphoma is one of the most frequently diagnosed cancers in the dog and multicentric LSA, characterized by peripheral lymph node enlargement, is the most common clinical presentation of this disease [18]. Often, patients are diagnosed with higher stage disease due to the fact that pet owners often have to recognize the lymph node enlargement before these pets are presented to a veterinarian for diagnosis [19]. The response to therapy for this disease is typically determined by serial measurements of peripheral lymph nodes. Inter- and intra-rater reliability of these measurements are reported to be good to excellent in the clinical setting [20]. Lymph node measurements are helpful in establishing the initial response to therapy evidenced by lymph nodes decreasing in size as well as determining disease progression when the lymph nodes increase in size following therapy. Monitoring peripheral lymph nodes as the primary indicator of treatment response is lacking as it does not detect minimal residual disease (MRD) after the lymph nodes have returned to a normal size. Previous studies in both humans and dogs have shown a variable amount of MRD following a positive response to therapy and the level of MRD at the end of a chemotherapy protocol has been shown to be prognostic [21, 22]. Lymph node measurements also fail to detect early indicators of disease progression as the disease burden must advance enough to cause lymph node enlargement before the patient is determined to be out of remission. Establishing liquid biopsy techniques, such as measuring nucleosome levels, in canine lymphoma could be helpful by providing objective measures of disease progression or treatment response even if the lymph nodes are normal in size. Such techniques could potentially reveal the MRD as well as provide an early indication of progressive disease prior to detectable lymph node enlargement.

The Nu.Q™ H3.1 Assay detects circulating nucleosomes in the blood of humans that occur with various disease states and has been used to detect and identify cancer even at early stages [13, 23]. This platform is an enzyme-linked immunosorbent assay (ELISA) directed at the histone H3.1 (H3.1) core histone protein. Previous investigations with histone H3.1 have identified cancer-associated mutations which induced nucleosome instability and enhanced cancer cell colony formation [24].

The objectives of this study were to determine whether H3.1 nucleosome concentrations could be used to distinguish healthy dogs and dogs with of LSA using the Nu.Q™ H3.1 assay as well as how nucleosome levels varied across disease stage and immunophenotype.

## Results

### Patient population

A total of 260 dogs were included in this study with 134 in the healthy control cohort and 126 in the LSA cohort. All healthy dogs and 10 dogs with lymphoma were recruited from the Texas A&M University Veterinary Medical Teaching Hospital (TAMU VMTH). The remaining 116 lymphoma samples were collected from the National Cancer Institute Division of Cancer Treatment and Diagnosis (NCI-DCTD) Canine Tumor Repository and represented 8 different collection sites. Information regarding the number of cases collected at each site along with number of cases per stage and immunophenotype are provided in Table 1. The healthy control cohort ranged in age from 0.83 to 14 years (median 6 years) and the LSA cohort ranged from 2 to 15 years (median 9 years). The healthy control cohort ranged in weight from 2.5 to 55.8 kg (median 23 kg) and the LSA cohort ranged from 5.0 to 74.5 kg (median 28.8 kg). The breeds most prevalent in the healthy control cohort were mixed breed (*n* = 29), Labrador retriever (*n* = 15), Australian cattle dog (*n* = 10), pit bull terrier (*n* = 7), border collie (*n* = 6), golden retriever (*n* = 5), dachshund (*n* = 4), and German shepherd (*n* = 3). The breeds most prevalent in the LSA population were mixed breed (*n* = 39), Labrador retriever (n = 10), cocker spaniel (n = 4), golden retriever (*n* = 8), Shetland sheepdog (n = 4), giant schnauzer (n = 3), and 2 or fewer of a variety of other pure bred dogs such as German Shepherd dogs, boxers, Basset Hounds and terriers. The healthy control cohort had a male to female ratio of 1.05 and a sex distribution including 4 intact females, spayed females (*n* = 61), intact males (n = 3), and castrated males (*n* = 65). Comparisons were made within the healthy cohort for age, body weight and gender and no statistical difference was found in between any of the groups (data not shown) [25]. The LSA cohort had a male to female ratio of 1.86 (82 males and 44 females) and a sex distribution including intact females (n = 3), spayed females (*n* = 41), intact males (*n* = 9), and castrated males (*n* = 73).

**Table 1:**
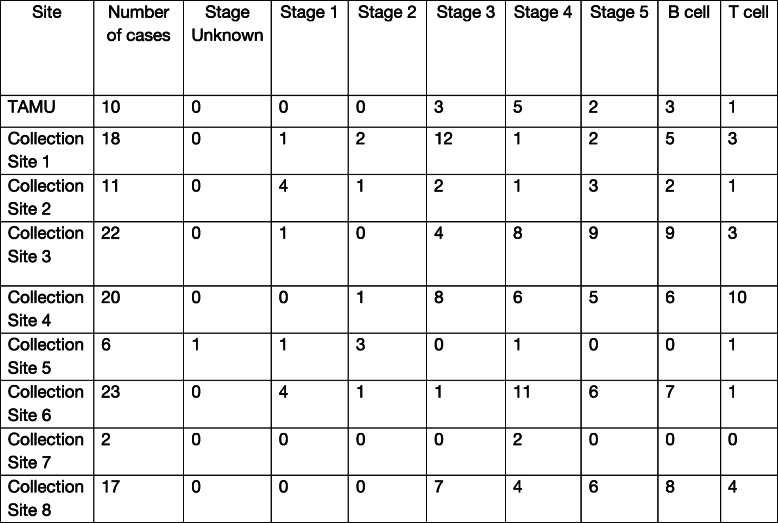
Number of cases per stage and immunophenotype for each collection site

### Nucleosome concentration

**Fig. 1:**
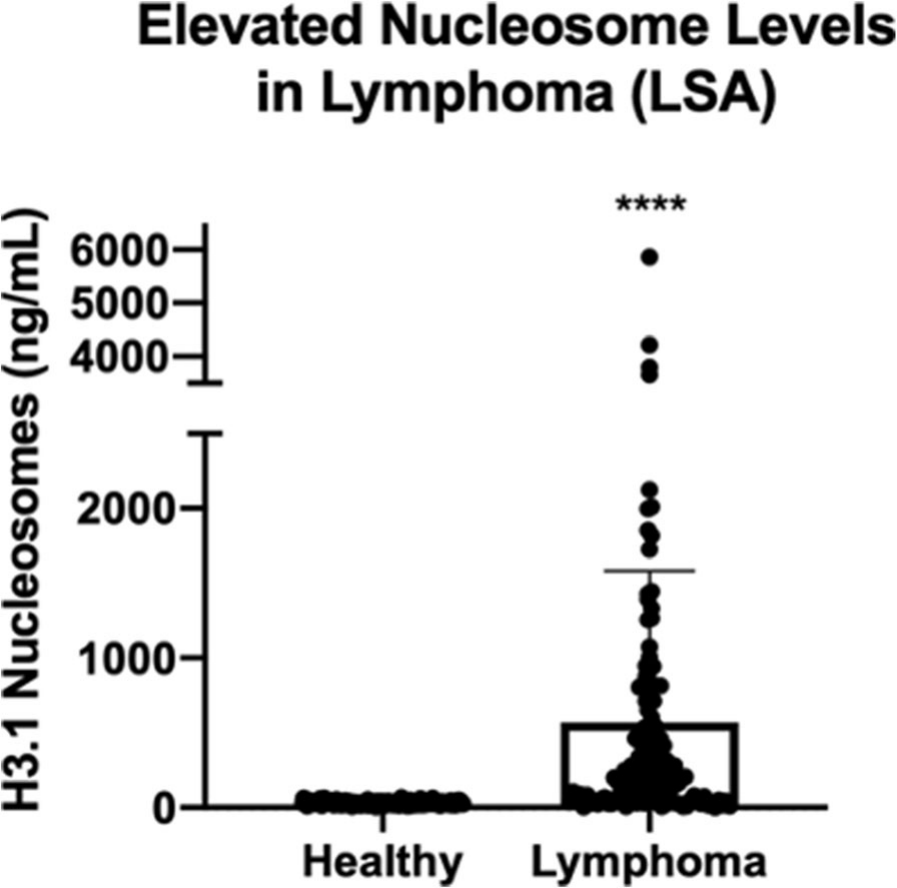
Elevated Nucleosome Levels in LSA. Mean plasma nucleosome concentrations (ng/mL) were significantly higher in LSA dogs compared to healthy controls. Boxes represent the mean and the bars represent the standard deviation. Dots represent individual data points. **** indicates a *p*-value < 0.0001

The nucleosome concentrations in the LSA cohort (median 211.1 ng/ml, mean 570.9 ng/mL, SEM 90.85, range 0–6544 ng/mL) were significantly higher than those in the healthy control cohort (median 31.1 ng/ml, mean 32.07 ng/mL, SEM 1.118, range 0–67.42 ng/mL) with a *p-*value of < 0.0001 (Fig. 1). According to the receiver operator characteristic (ROC) curve the area under the curve was 87.8% with a sensitivity of 80.16% and a specificity of 94.78% with a cut off for the healthy range set at 67.5 ng/mL (nucleosome range for all healthy dogs was 6.33–67.42 ng/mL).

Comparisons were made between nucleosome concentrations for LSA patients from each collection site. The median nucleosome concentration for samples collected at TAMU-VMTH was 429.5 ng/ml (n = 10, mean 818 ng/ml, SEM 198, range 32.2–4218 ng/ml), at collection site 1 was 219.3 ng/ml (*n* = 18, mean 495.1 ng/ml, SEM 188.5, range 0–2689 ng/ml), at collection site 2 was 154.8 ng/ml (*n* = 11, mean 597.8, SEM 284, range 38.1–2450 ng/ml), at collection site 3 was 164.4 ng/ml (*n* = 22, mean 604 ng/ml, SEM 188.4, range 0–2451 ng/ml), at collection site 4 was 247.3 (*n* = 20, mean 535.8 ng/ml, SEM 150.9, range 23.3–2450 ng/ml), at collection site 6 was 193.8 ng/ml (*n* = 23, mean 193.8, SEM 61.9, range 0–958.5 ng/ml), and at collection site 8 was 177.4 ng/ml (*n* = 17, mean 421.3, SEM 121.6, range 19.5–1730 ng/ml). Collection sites 5 and 7 did not have sufficient case numbers for meaningful comparisons (6 and 2 cases, respectively). No significant differences were found for nucleosome concentration between collection sites with the *p*-value for all comparisons > 0.999.

Nucleosome concentrations were evaluated in LSA patients to determine if they were affected by age, gender, or body weight. Information for sex was available for all LSA dogs and information for age and body weight were available for 120 LSA dogs. LSA dogs were split into 3 age groups (1–5 years *n* = 27, 6–10 years *n* = 70, and > 10 years n = 23). There were no statistically significant differences in nucleosome concentration among the different age groups (Table 2). Dogs with LSA were split into 4 different groups based on gender (female spayed n = 41, female intact n = 3, male neutered n = 73, and male intact n = 9) and no statistically significant differences were found between these groups (Table 3). Finally, the LSA dogs were split into 4 groups based on body weight (< 15 kg n = 29, 15–30 kg n = 41, 31–45 kg n = 41, and > 45 kg n = 9). No statistically significant differences were identified between different body weight groups (Table 4).

**Table 2:**
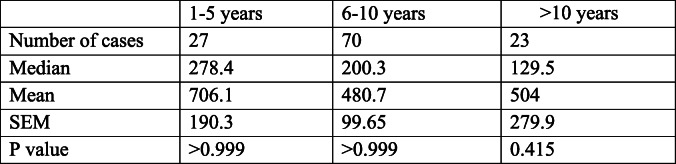
Nucleosome concentrations in dogs with lymphoma separated by age

**Table 3:**
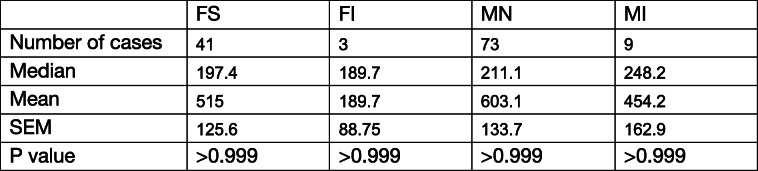
Nucleosome concentrations in dogs with lymphoma separated by gender

**Table 4:**
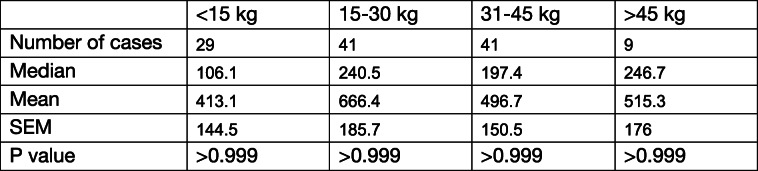
Nucleosome concentrations in dogs with lymphoma separated by body weight

**Fig. 2:**
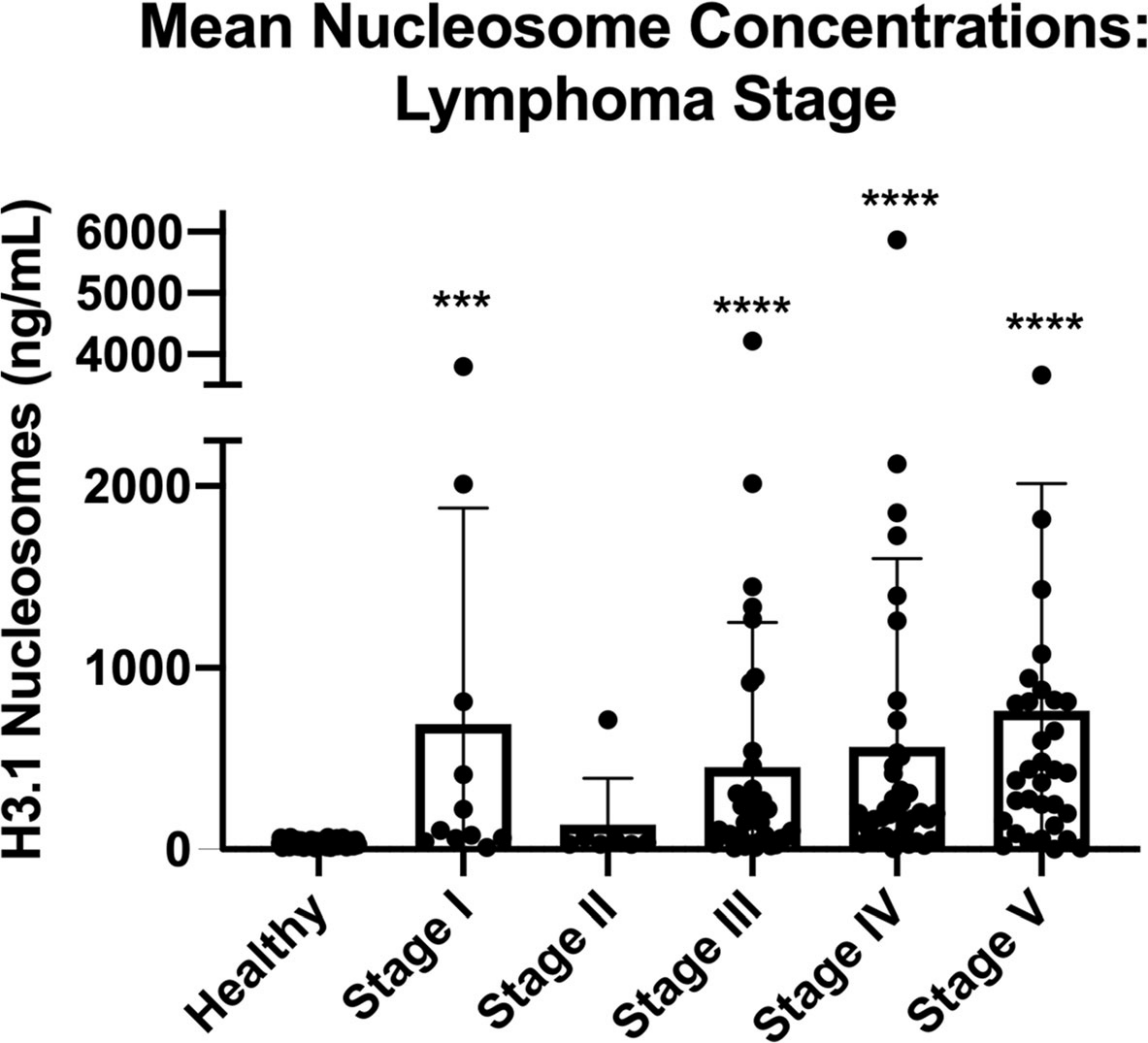
Mean Nucleosome Concentrations: LSA by Stage. Mean plasma nucleosome concentrations (ng/mL) in all LSA stages (except stage II) were significantly higher than healthy controls. Boxes represent the mean and the bars represent the standard deviation. Dots represent individual data points. *** indicate a p-value < 0.001, **** indicate a p-value < 0.0001

To determine whether nucleosome concentrations were elevated across all stages of LSA, we compared nucleosome concentrations between healthy controls and the different stages of lymphoma. Stage of disease was available for all patients included in this study. All stages of LSA, except stage II, had significantly elevated nucleosome concentrations compared to healthy controls (Fig. 2). The median nucleosome concentration for stage I LSA was 104.9 ng/ml (n = 11, mean 691.9 ng/ml, SEM 358, p-value 0.0002, AUC 87.99%), for stage II LSA was 36.2 ng/ml (n = 7, mean 135.6 ng/ml, SEM 96.45, p-value > 0.088, AUC 69.2%), for stage III LSA was 177.5 ng/ml (*n* = 37, mean 452.9 ng/ml, SEM 130.4, p-value < 0.0001, AUC 85.1%), for stage IV LSA was 200.2 ng/ml (*n* = 38, mean 564.2 ng/ml, SEM 167.9, p-value < 0.0001, AUC 91.7%), and for stage V LSA was 421.4 ng/ml (*n* = 33, mean 763.0 ng/mL, SEM 217.7, p-value < 0.0001, AUC 90.3%).

**Fig. 3:**
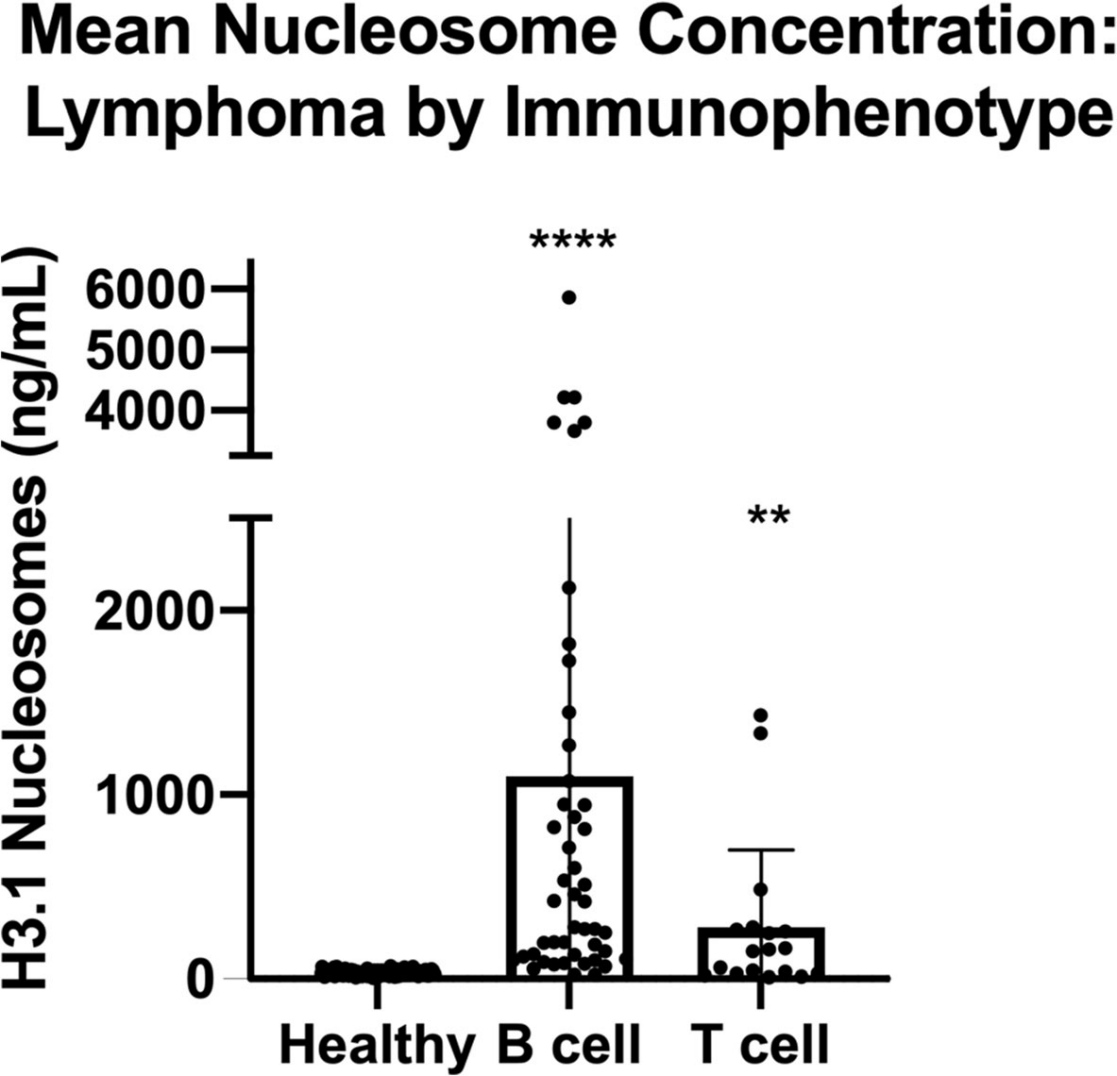
Mean Nucleosome Concentrations: LSA by Immunophenotype. Mean plasma nucleosome concentrations (ng/mL) in B- and T-cell LSA were significantly higher than healthy controls. B-cell LSA mean nucleosome concentrations were significantly higher than T-cell LSA. Boxes represent the mean and the bars represent the standard deviation. Dots represent individual data points ** indicate a p-value < 0.01, **** indicate a p-value < 0.0001

We next investigated whether elevated nucleosome concentrations were common to both B-cell and T-cell LSA. Immunophenotyping information was available for 61 LSA cases, and nucleosome concentration was compared amongst two immunophenotype groups and healthy controls. Nucleosome concentrations were significantly elevated in both B-cell and T-cell LSA compared to healthy controls (Fig. 3). The median nucleosome concentration for B-cell LSA was 421.42 ng/ml (*n* = 43, mean 1031.7 ng/ml, SEM 234.2, p-value < 0.0001, AUC 98%) and 153.7 ng/ml for T-cell LSA (n = 18, mean 277.6 ng/ml, SEM 99.4 p-value 0.0006, AUC 74.9%). T-cell LSA patients were found to have a significantly lower nucleosome concentration than B-cell LSA patients (p-value 0.018). In the B cell lymphoma cohort there was one dog with WHO stage I disease (2.3%), no dogs with stage II disease, 13 dogs with stage III disease (30.2%) and 15 dogs each with stage IV (34.9%) and 14 with stage V disease (32.6%). For the T cell lymphoma cohort there were no dogs with stage I disease, one dog with stage II disease (5.6%), 10 dogs with stage III disease (55.6%), 2 dogs with stage IV disease (11.1%) and 5 dogs with stage V disease (27.8%). When using the compressed WHO staging system previously published by Valli et al. in 2013, the two have a similar distribution of stage with compressed stage 1 (stages I/II) including one dog each (B cell 2.3% and T cell 5.5%), stage 2 (compressed stages III/IV) the B cell cohort had 28 cases (65.1%) and the T cell cohort had 12 cases (66.6%), finally for the compressed stage 3 (stage V) the B cell cohort had 14 (32.5%) cases and the T cell cohort had 5 cases (27.8%) [19].

**Fig. 4:**
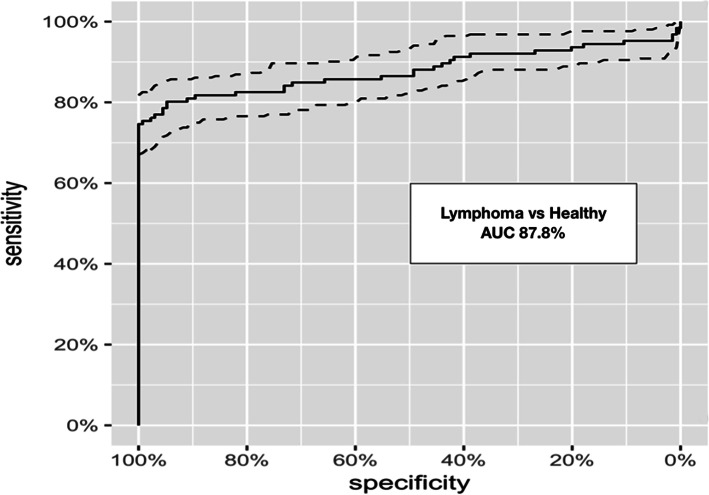
Discriminating LSA from Healthy Controls. ROC analysis with an established threshold of 67.5 ng/ml generated an area under the curve of 0.878. This threshold produced a sensitivity of 80.16% at a specificity of 94.78%

A receiver operating characteristic analysis was performed with an established threshold of 67.4 ng/ml which generated an area under the curve of 0.878 (Fig. 4). This threshold produced a sensitivity of 74% at a specificity of 100%. The performance of this threshold for each specific stage was investigated by applying it retroactively to the population of LSA patients. This analysis showed that the threshold could accurately distinguish LSA patients from healthy patients in 63% (7/11) of stage I patients, 14.3% (1/7) of stage II patients, 75.7% (28/37) of stage III patients, 81.6% (31/38) of stage IV patients, and 81.8% (27/33) of stage V patients. Performance was also evaluated by immunophenotype and the threshold could distinguish LSA patients from healthy patients in 95.3% (41/43) of B-cell LSA and 55.6% (10/18) of T-cell LSA.

## Discussion

The current study follows from previous findings in which nucleosome levels helped with early detection of cancer in humans and were significantly elevated in a small cohort of dogs with LSA [13, 17]. As with most studies, there were limitations present in this study. Samples received from the DCTD Canine Tumor Repository had variable amounts of patient demographic, staging, treatment and outcome data available which would have been useful in generating more power for the analysis and better characterization of those cases with low stage disease and T cell phenotypes. Additionally, the samples derived from the DCTD Canine Tumor Repository had been stored much longer than those collected from TAMU, potentially up to 5 years or more. Though not statistically different, the TAMU lymphoma cases (*n* = 10) had higher plasma nucleosome concentrations than those collected from the biobank. A study by Holdenrieder et al. determined that there is some loss over time of nucleosomes in EDTA stabilized serum samples that is slightly less than 7% per year [26]. This likely explains the differences in the median concentrations from those collected at TAMU and those from the biobank. However, all healthy canine samples were also collected prospectively at TAMU and stored for less than 6 months. The lack of variability and low median and mean plasma concentrations in the 134 healthy dog samples regardless of age, body weight or gender (median 31.1 ng/ml, mean 32.07 ng/mL, SEM 1.118, range 0–67.42 ng/mL) adds credence to the utility of this test to discriminate between healthy dogs and those with lymphoma. Additionally, it is possible that the reported sensitivity is lower than what it is actually true for this test if there was loss of nucleosomes in storage. Shorter storage times may have resulted in a larger difference between the healthy and LSA cohorts, and, subsequently, an improved discrimination between the two groups than what was reported in this study. Lastly, the healthy control population was screened with a physical examination and questionnaire regarding the pet’s health status. Biochemical analysis such as a complete blood count, serum biochemistry, and urinalysis were not performed on this population, and it is possible that an underlying silent disease process may not have been discovered during healthy patient evaluation.

As previously described by this group in a small cohort of dogs with lymphoma, nucleosome concentrations were significantly elevated in the cohort of LSA patients when compared to the healthy control cohort [17]. The median plasma nucleosome concentration in LSA patients was 6.8 times higher than in the healthy controls. Broadly we found that elevated nucleosome concentrations were present at all cancer stages, except stage II, and present in both B and T cell lymphoma. There were no significant differences found when comparing nucleosome concentrations in lymphoma patients by age, gender, or body weight groups.

When evaluated by stage, only the dogs with stage II LSA were found to not have significant elevations compared to healthy controls. This subpopulation contained only 7 dogs and the lack of significance is suspected to be due to a population of insufficient size. This was supported by a post-hoc power analysis with an alpha set at 0.05 and a power set at 80% which indicated that a minimum of 16 cases would be needed to effectively evaluate this group. Another potential consideration is that the tumor burden associated with this stage of disease does not produce more nucleosomes than healthy dogs. However, this is unlikely since the nucleosome concentrations of dogs diagnosed with stage I LSA were significantly elevated compared to healthy controls in this study. Another consideration for the low nucleosome concentration in the stage II LSA cases is a T cell phenotype. The dogs in this study with T cell LSA had significantly lower nucleosome concentrations than those with B cell LSA, however, upon further review, only one of the dogs with stage II LSA had immunophenotype data available and this dog did, indeed, have T cell LSA. Additional collection and analysis of lower stage LSA patients with full characterization of their disease is needed to help further characterize the nucleosome concentrations in these patient populations.

As mentioned above, while both B-cell and T-cell LSA had significant increases in nucleosome concentration compared to healthy controls, B-cell LSA patients had a 2.7 fold higher median nucleosome concentration as compared to T-cell LSA patients. The underlying mechanism of this difference is unknown. One potential explanation is that while T-cell LSA patients often have peripheral lymphadenopathy, it is the authors’ experience that their disease burden is subjectively lower than their B-cell counterparts in the clinical setting. The lower nucleosome concentration detected in this study may be the result of an overall lower disease burden that occurs between B-cell and T-cell LSA. In humans, it has been shown that the amount of cfDNA shed by a LSA patient depends on the particular LSA subtype [27]. It is possible this is also true in dogs and the difference between B-cell and T-cell LSA nucleosome concentrations are indicative of underlying pathophysiologic differences between these LSA subtypes. Finally, owing to the fact that many samples for the lymphoma cohort were purchased from a biobank, most of the cases in this population were not characterized by flow cytometry. It is possible that some of the samples in this group were from dogs with indolent T-cell LSA. Standard immunophenotyping (CD3 positivity) would not be able to differentiate the less aggressive T-cell lymphomas from the more aggressive T cell lymphomas. Studies in humans have also shown that the levels of cfDNA are higher in more aggressive subtypes of LSA [27, 28]. If indolent LSA cases were included in the population of T-cell LSA cases, they may have artificially lowered the overall nucleosome concentration in this population.

A sensitivity of 80.16% at a specificity of 94.78% in distinguishing LSA patients from healthy controls was achieved using nucleosome concentrations with a threshold of 67.5 ng/ml. This indicates that nucleosomes could be a useful screening tool in the differentiation of dogs with LSA from healthy dogs. The ROC curve demonstrated that some cases of LSA fell below the discrimination line. These cases were of lower stage or had a T cell phenotype and had plasma nucleosome concentrations similar to the healthy control cohort. This is to be expected as nucleosome concentrations are correlated with stage and, therefore, tumor burden in humans [13, 29, 30]. Similar results were found in the dogs evaluated in this study where the nucleosome concentration increased with stage and tumor burden. Despite this finding, the established threshold was successful in discriminating 63.6% of stage 1 LSA patients from healthy controls. This is an encouraging finding as it shows that circulating nucleosomes could be used as a tool for early disease detection and could be helpful when a diagnosis is difficult to establish.

## Conclusion

The results of this study demonstrate that plasma nucleosome concentrations of dogs with LSA are significantly elevated compared to healthy controls. These findings support the use of nucleosomes as a tool for the early detection of LSA in dogs.

## Methods

### Healthy dogs

Dogs were recruited from patients presenting to the TAMU VMTH for routine wellness exams or from dogs owned by TAMU VMTH personnel. All animal studies were approved by the Texas A&M University Animal Care and Use Committee (AUP #2017–0350). Owners were questioned to determine the health status of each patient and a physical exam was performed at the time of sample collection. In order to be eligible for inclusion, dogs were required to be over one year of age and healthy. Dogs were excluded if there was any secondary significant inflammatory/infectious disease or previous diagnosis of neoplasia. A disease was considered significant if the dog was currently undergoing therapy or if the disease was expected to alter the dog’s survival. Dogs were allowed to participate if they were on joint supplements, flea and tick preventative and/or heart worm prevention. Information recorded for each patient included signalment, body weight, body condition score, and any relevant comorbidities reported by the owner.

### Lymphoma dogs

The LSA dog cohort was recruited in part from dogs presenting to the TAMU VMTH for treatment of naive multicentric LSA (AUP #2019–0211). For the TAMU VMTH patients, the diagnosis of lymphoma was made either through the use of lymph node cytology, flow cytometry, or a combination of these techniques. The remaining samples were recruited from the National Cancer Institute Division of Cancer Treatment and Diagnosis (NCI-DCTD) Canine Tumor Repository. Samples provided to the NCI-DCTD were collected prior to the initiation of therapy, and were confirmed to be lymphoma via histopathology, immunophenotyping data, and/or cytologic diagnosis provided to the repository. When available, information including patient signalment, body weight, body condition score, stage of disease, and immunophenotype were recorded.

### Sample collection and processing

For patients presenting to the TAMU VMTH, 3–5 mLs of blood was collected from dogs fasted at least 6 h before collection and immediately placed in K2-EDTA blood collection tubes. Within one hour of collection, samples were centrifuged at room temperature at 3000×g for 10 min. Plasma was then immediately removed without disrupting the buffy coat layer, placed in pre-labeled cryovials and frozen at − 80 °C to run in batches. Processing samples with this protocol was shown to be appropriate for reliable, consistent nucleosome detection in dog plasma [17]. Samples received from the DCTD Canine Tumor Repository were stored frozen at − 80 °C to be run in batches. For these collections, approximately 25–30 mLs of blood and 2–3 mLs of urine were collected before surgical excision or biopsy of treatment naïve tumor tissue. Body fluids were processed and stored at −80C within one hour of collection. The initial centrifugation and storage requirements were in line with what is required by the assay. Plasma samples stored at -80 °C for DNA analysis have been shown to be stable for more than 10 years and nucleosomes concentrations in EDTA stabilized serum samples have been shown to have minimal loss (< 7% per year) after long term storage (> 60 months) [26, 31].

### Nucleosome assays

Frozen samples were thawed and allowed to come to room temperature for at least 30 min prior to analysis. All samples were performed in duplicate. The samples were evaluated using the Nu.Q™ H3.1 ELISA (Belgian Volition, SRL, Isnes, Belgium) and were performed according to the manufacturer’s instructions. Briefly, a standard curve was generated using the known standards provided. Before use, the wells were washed 3 times with 200 μL of the provided diluted wash solution with excess solution being removed after each wash. Patient and healthy dog plasma samples were vortexed and then centrifuged for 2 min at 11,000×g at 4 °C before samples were loaded into the plates. Lymphoma samples were diluted 3-fold in order to ensure that they would register on the plates within the limits of the colorimetric standards. Twenty microliters of patient samples and kit controls were run in duplicate in wells on 96 well plates. Eighty microliters of assay buffer was then added to each well. The plates were sealed with foil and incubated at room temperature for 2.5 h under agitation at ~ 700 rpm. Plates were emptied and washed as described above. Next, 100 μL of HRP labelled detection antibody was added to each well. The plate was sealed with foil and incubated at room temperature for 1.5 h under agitation at ~ 700 rpm. Plates were then emptied and washed as described above. Next, 100 μL of TMB substrate was added to each well. The plate was sealed with foil and incubated at room temperature for 20 min in the dark under agitation at ~ 700 rpm. One hundred microliters of stop solution were then added and the plate was shaken gently. Plates were read at an absorbance of 450 nm (BioTek Synergy H1 plate reader, BioTek Instruments, Winooski, VT) within 5 min of stop solution being added. The standard curve was linearized and fitted to a 5-parameter logistic curve using statistical software (Graphpad Software, version 8, San Diego, CA).

### Statistical analysis

Descriptive statistics for the patient populations were performed using Microsoft excel for Mac (v. 16.16.27, 2016). For data sets containing only two cohorts, such as the healthy controls versus all LSA cases, a Wilcoxon rank sum test was used to compare the medians of the data sets. For data sets where multiple conditions were compared such as disease stage, a two-way ANOVA for repeat measures with a Tukey’s multiple comparisons test was performed. This part of the analysis was performed using GraphPad Prism version 8.0.0 for Macintosh, GraphPad Software, San Diego, California USA, www.graphpad.com. Spearman’s correlation, ROC curves and specificity/sensitivity calculations were performed using R version 3.4.3 and the pROC package [32, 33].

## Data Availability

All relevant data are within the paper.

## Abbreviations

LSA: Lymphoma
TAMU VMTH: Texas A&M University Veterinary Medical Teaching Hospital
ROC: Receiver Operating Characteristic
MRD: Minimal Residual Disease
